# Network alterations underlying anxiety symptoms in early multiple sclerosis

**DOI:** 10.1186/s12974-022-02476-0

**Published:** 2022-05-24

**Authors:** Erik Ellwardt, Muthuraman Muthuraman, Gabriel Gonzalez-Escamilla, Venkata Chaitanya Chirumamilla, Felix Luessi, Stefan Bittner, Frauke Zipp, Sergiu Groppa, Vinzenz Fleischer

**Affiliations:** 1 Department of Neurology, Focus Program Translational Neuroscience (FTN), Rhine Main Neuroscience Network (rmn2), University Medical Center, Johannes Gutenberg University Mainz, Mainz, Germany; 2 Biomedical Statistics and Multimodal Signal Processing Unit, Department of Neurology, Focus Program Translational Neuroscience (FTN) Neuroimaging Center, Rhine Main Neuroscience Network (rmn2), University Medical Center, Johannes Gutenberg University Mainz, Langenbeckstr. 1, 55131 Mainz, Germany; 3 Section of Movement Disorders and Neurostimulation, Department of Neurology, Focus Program Translational Neuroscience (FTN), Rhine Main Neuroscience Network (rmn2), University Medical Center, Johannes Gutenberg University Mainz, Mainz, Germany

**Keywords:** Multiple sclerosis, Anxiety, Atrophy, Functional connectivity, Excitability

## Abstract

**Background:**

Anxiety, often seen as comorbidity in multiple sclerosis (MS), is a frequent neuropsychiatric symptom and essentially affects the overall disease burden. Here, we aimed to decipher anxiety-related networks functionally connected to atrophied areas in patients suffering from MS.

**Methods:**

Using 3-T MRI, anxiety-related atrophy maps were generated by correlating longitudinal cortical thinning with the severity of anxiety symptoms in MS patients. To determine brain regions functionally connected to these maps, we applied a technique termed “atrophy network mapping”. Thereby, the anxiety-related atrophy maps were projected onto a large normative connectome (*n* = 1000) performing seed‐based functional connectivity. Finally, an instructed threat paradigm was conducted with regard to neural excitability and effective connectivity, using transcranial magnetic stimulation combined with high-density electroencephalography.

**Results:**

Thinning of the left dorsal prefrontal cortex was the only region that was associated with higher anxiety levels. Atrophy network mapping identified functional involvement of bilateral prefrontal cortex as well as amygdala and hippocampus. Structural equation modeling confirmed that the volumes of these brain regions were significant determinants that influence anxiety symptoms in MS. We additionally identified reduced information flow between the prefrontal cortex and the amygdala at rest, and pathologically increased excitability in the prefrontal cortex in MS patients as compared to controls.

**Conclusion:**

Anxiety-related prefrontal cortical atrophy in MS leads to a specific network alteration involving structures that resemble known neurobiological anxiety circuits. These findings elucidate the emergence of anxiety as part of the disease pathology and might ultimately enable targeted treatment approaches modulating brain networks in MS.

**Supplementary Information:**

The online version contains supplementary material available at 10.1186/s12974-022-02476-0.

## Introduction

Multiple sclerosis is a demyelinating autoimmune disease of the CNS leading to disability in young adults. Sensory and motor deficits are characteristic symptoms, but also cognitive and neuropsychiatric symptoms can occur, even in early disease stages [1–4]. With regard to affective symptoms, anxiety is considered a major debilitating symptom in multiple sclerosis, impairing quality of life [5–7]. Moreover, a large number of patients develop anxiety symptoms years before clinical disease manifestation or motor symptoms [8]. Interestingly, anxiety, although often still seen as co-morbidity, is however associated with the long-term development of cognitive impairment and memory deficits [6, 9]. Cognitive deficits, in turn, are clearly related to neurodegeneration [10]. Therefore, early neurodegenerative processes in multiple sclerosis might also cause anxiety symptoms. However, MRI-derived total brain volume as well as white matter lesion load was not associated with anxiety levels in multiple sclerosis in early association studies [11, 12]. More recent evidence from smaller scope studies suggests that some regional volumetric associations may exist. In particular, multiple sclerosis patients with fatigue and increased anxiety had larger caudate volumes and a thinner left parietal cortex compared to those without fatigue [13]; another study revealed that anxiety in multiple sclerosis may have a neuropathological substrate in the septo-fornical area [14]. However, the lack of a robust association between anxiety symptoms and structural MRI abnormalities may have led to the opinion that anxiety is rather a reactive response of patients facing a chronic disease.

Both functional MRI (fMRI) and EEG are valuable techniques to depict brain functional connectivity between distant brain regions. Apart from animal data [15], experimental human data derived from fMRI and EEG point towards the involvement of impaired excitability and network desynchronization in multiple sclerosis [16–18]. In particular, cognitive impairment has been linked to functional network disturbances in multiple sclerosis patients [19]. Applying EEG, it was reported that multiple sclerosis patients show an increased excitability of frontotemporal regions and decreased coherence of short and long distance connections at rest in relation to cognitive impairment [16]. Using fMRI, one study displayed enhanced regional activation within the ventrolateral prefrontal cortex (PFC) and a lack of functional connectivity between the PFC and the left amygdala in multiple sclerosis patients when exposed to emotional stimuli [20]. In addition, resting state brain networks, particularly the default mode network, have been found to be altered in several psychopathological conditions such as anxiety [21–25]. Studies combining structural and functional neuroimaging data in multiple sclerosis patients have demonstrated that thalamic atrophy is associated with disruption of cortical functional networks and is related to worse cognitive function [26–28]. However, studies investigating the neural correlates of anxiety symptoms in multiple sclerosis patients integrating structural and functional imaging approaches are surprisingly missing.

“Atrophy network mapping” is a new technique that performs seed-based functional connectivity using a normative functional connectome to determine brain regions functionally connected to atrophy patterns. This approach has recently lent insight into the localization of neuropsychiatric symptoms in neurodegenerative disorders [29–31].

Our goal in this study was to identify anxiety-underlying network changes and their structure–function association in multiple sclerosis. Cortical atrophy maps were related to the severity of anxiety symptoms in multiple sclerosis patients and projected onto a large (*n* = 1000) normative resting-state functional MRI connectome. Structural equation modeling (SEM) was then applied to determine the causal relation between anxiety symptoms and the MRI volumes of the brain regions belonging to the detected functional network. To confirm our main findings, we furthermore investigated functional connectivity and cortical excitability in an additional cohort, applying transcranial magnetic stimulation and high-density electroencephalography (TMS–HD-EEG), both at rest and during an instructed threat paradigm in multiple sclerosis patients and controls [32–34].

## Methods

### Subjects

**Fig. 1:**
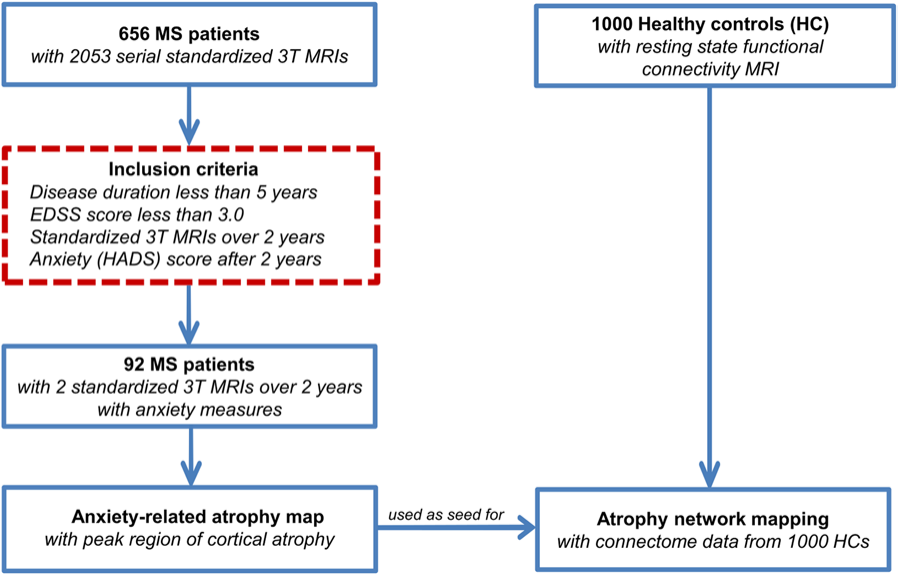
Study design and work flow. After fulfilling the inclusion criteria (red dashed line box), 92 early multiple sclerosis patients were included for the cortical thickness correlation analysis with HADS-A scores (anxiety symptoms) to generate an anxiety-related atrophy map. Next, atrophy network mapping was applied to project this atrophy map onto a normative functional brain network (*n* = 1000). As a result, we were able to determine brain regions functionally connected to the atrophied location

Out of a cohort of 656 multiple sclerosis patients with standardized MRIs from 2011 to 2017, 92 early clinically isolated syndrome/relapsing–remitting multiple sclerosis patients with additional anxiety measures were eligible and included in this study (Fig. 1 and Table 1). These multiple sclerosis patients [63 female, mean age ± SD: 34.4 ± 9.5 years, mean disease duration: 1.9 ± 3.4 years, mean Expanded Disability Status Scale (EDSS) ± SD: 1.2 ± 1.1] underwent MRI twice over a study period of 2 years (mean follow-up time ± SD: 2.4 ± 1.4 years). In addition, patients were clinically assessed in our outpatient clinic by an experienced neurologist to determine the EDSS score. The EDSS is a clinician-administered assessment scale evaluating the functional systems of the central nervous system [35]. EDSS scores were assessed 30 days after a relapse onset. For inclusion in our study, the EDSS had to be below 3.0 and the disease duration less than 5 years. These thresholds were chosen to guarantee a mildly affected multiple sclerosis cohort without considerable motor impairment. Moreover, clinical relapses and radiological disease activity [to establish “no evidence of disease activity” (NEDA-3)] were assessed. The self-administered anxiety score Hospital Anxiety and Depression Scale-Anxiety subscale (HADS-A), which is a tool used to screen for the presence of anxiety [36, 37], was determined after 2 years. The questionnaire was filled out by the patient and returned to the clinician.

**Table 1:** Clinical data of the multiple sclerosis patient cohort

Demographic and clinical data	Multiple sclerosis patients (*n* = 92)		
Sex (female/male)	62/30		
Disease course at baseline (CIS/RRMS)	21/71		
Mean age at baseline MRI (SD) [years]	34.4 ± 9.5		
Mean age at disease onset (SD) [years]	32.5 ± 9.5		
Mean disease duration (SD) [years]	1.9 ± 3.4		
DMD (no/first line/second line)^c^	24/54/14		
Mean follow-up (SD) [years]	2.4 ± 1.4		
Mean EDSS score (SD)	1.2 ± 1.1		
Mean HADS-A score (SD) after 2 years	5.5 ± 4.1		

Volumetric analysis	Baseline	Follow-up	*p* value
Mean GM volume (SD) [ml]	632 ± 629	614 ± 607	0.001^a^
Mean TB volume (SD) [ml]	1441 ± 1411	1436 ± 1411	0.008^a^
Median T2 WM lesion volume (range) [ml]	1.5 (0.1–83.2)	2.0 (0.1–124.3)	0.001^b^

Demographic and clinical data as well as brain volumetric measurements of early-stage multiple sclerosis patients at baseline MR scan and after follow-up

*CIS* clinically isolated syndrome, *RRMS* relapsing–remitting multiple sclerosis, *SD* standard deviation, *EDSS* Expanded Disability Status Scale, *GM* grey matter, *TB* total brain, *DMD* disease-modifying drugs, *HADS-A* Hospital Anxiety and Depression Scale-Anxiety subscale

^a^*p* values derived from paired *t* test

^b^*p* values derived from Wilcoxon signed-rank test

^c^First line: glatiramer acetate, interferon-beta, teriflunomide, dimethyl fumarate; second line: natalizumab, fingolimod, alemtuzumab

Moreover, 18 additional patients with relapsing–remitting multiple sclerosis (10 female, mean age ± SD: 36.8 ± 9.4 years, mean disease duration 3.9 ± 5.0 years, mean EDSS ± SD: 2.2 ± 1.4) and 18 healthy controls (9 female, mean age: 36.0 ± 9.0 years) were selected to participate in a TMS–HD-EEG study at rest and under task in addition to the HADS-A assessment and structural MRI acquisition (Additional file 1: Table S1).

Resting-state fMRI data from 1000 healthy individuals (58% female, age range between 19 and 35 years; mean age 21.5 years) freely available from the normative database of the Brain Genomics Superstruct Project (GSP) [38] were used to link atrophy to a common brain network (see below).

The local ethics committee of the medical faculty of the Johannes Gutenberg University Mainz (Mainz, Germany) approved the study protocol, which is according to the Declaration of Helsinki; all participants provided written informed consent.

### MRI data acquisition

Structural MRI was performed on a 3-T MRI scanner (Magnetom Tim Trio, Siemens, Germany) with a 32-channel receive-only head coil. In all patients, imaging was performed using a sagittal 3D T1-weighted magnetization-prepared rapid gradient echo (MP-RAGE) sequence (TE/TI/TR = 2.52/900/1900 ms, flip angle = 9°, field of view = 256 × 256 mm^2^, matrix size = 256 × 256, slab thickness = 192 mm, voxel size = 1 × 1 × 1 mm^3^) and a sagittal 3D T2-weighted fluid-attenuated inversion recovery (FLAIR) sequence (TE/TI/TR = 388/1800/5000 ms, echo-train length = 848, field of view = 256 × 256 mm^2^, matrix size = 256 × 256, slab thickness = 192 mm, voxel size = 1 × 1 × 1 mm^3^). Major anatomical abnormalities were excluded by a clinician scientist blinded to the patient data based on the subject’s T1-weighted and FLAIR images of the whole brain.

### MRI preprocessing

**Fig. 2:**
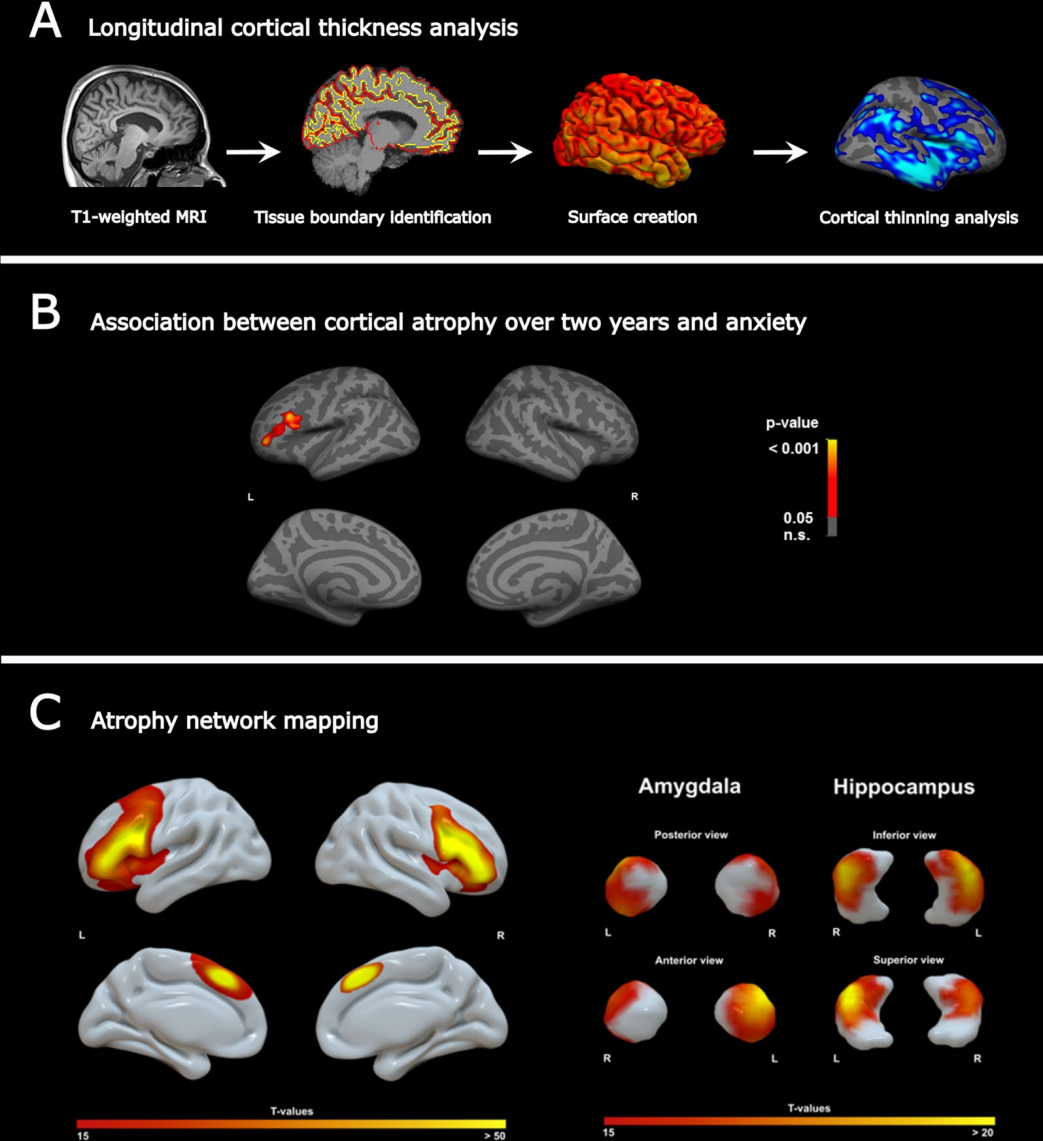
**A** Longitudinal cortical thickness analysis. Workflow for longitudinal MRI morphometric analysis. **B** Anxiety-related atrophy maps across multiple sclerosis patients. Atrophy of the left PFC (peak region: rostral middle frontal lobe) was associated with anxiety scores measured by the HADS-A scale. **C** Atrophy network mapping. Generated atrophy maps are used in a seed-based functional connectivity analysis in a large dataset of healthy controls to find functionally connected regions. The atrophy network mapping approach identified the PFC, amygdala and hippocampus as brain regions functionally connected to the previously detected atrophied location

MRI T1 images from all multiple sclerosis patients were preprocessed using FreeSurfer (v6.0; http://surfer.nmr.mgh.harvard.edu). In brief, the pipeline includes the removal of non-brain tissue and intensity normalization, followed by subcortical segmentation and cortical surface reconstruction via tessellation of the grey matter (GM)/white matter (WM) and GM/CSF boundaries, accompanied by automated topology correction with accurate surface deformation to identify tissue borders (Fig. 2A). Cortical thickness was then calculated as the distance between the WM and GM surfaces at each point (vertex) of the reconstructed cortical mantle [39]. Individual results were visually inspected to ensure accuracy of the surface creation. Errors in the surface reconstruction were manually corrected to improve the cortical thickness estimation. Given the longitudinal nature of the study, the resulting cross-sectional preprocessed data was then used to create a mean single-subject template, to which each time-point image was rigidly transformed. This final space transformation further reduces inter-individual variability and permits an implicit vertex correspondence across all time points [40]. After preprocessing, mean cortical thickness based on the Desikan−Killiany atlas [41] and mean subcortical volumes [42] were obtained for each region. From cortical thickness values, individual maps of cortical atrophy (annual atrophy, expressed in mm^3^ per year) were defined as: atrophy = (CT_Follow-up_ − CT_Baseline_)/(MRI time Δ). The CT_Baseline_ and CT_Follow-up_ are the estimated cortical thickness maps at each time point, and “MRI time Δ” is the individual delay between the two MRI scans in years. The same procedure was used to calculate atrophy of subcortical volumes.

### Functional MRI data and preprocessing

Normative resting-state fMRI data from the 1000 healthy subjects were acquired at Harvard Medical School and Massachusetts General Hospital and are part of the publicly available GSP dataset [38]. These fMRI data were obtained with a 3-T Tim Trio scanner (Siemens Healthcare, Erlangen, Germany) MRI using a 12-channel receive coil array scanner.

Resting-state fMRI data was acquired at 3 mm isotropic resolution with TR = 3000 ms and 124 frames. fMRI data preprocessing included (1) removal of the first five frames, (2) motion correction using rigid body translation and rotation, (3) slice timing correction, (4) alignment with structural image, (5) normalization to Montreal Neurological Institute (MNI) space using the deformation matrices obtained during MRI preprocessing with the CAT12 toolbox (Structural Brain Mapping group, Jena University Hospital, Jena, Germany), (6) smoothing by a 6 mm full-width half-maximum (FWHM) kernel, (7) nuisance covariate regression (including six motion correction parameters, and averaged WM and CSF signals), and (8) bandpass filtering (between 0.01 and 0.08 Hz). WM and CSF masks were obtained from segmentation of the anatomical T1 image, followed by binarizing the probabilistic tissue maps at a threshold of 0.9 and 0.7, respectively. All preprocessing steps were carried out following recommended guidelines using SPM12 [43].

### Atrophy network mapping

For the multiple sclerosis cohort, we derived a “functional network map”—defined as brain regions functionally connected to the previously generated anxiety-related atrophy map [30, 31]. To this end, we used FreeSurfer (v6.0; http://surfer.nmr.mgh.harvard.edu) to determine, in a vertex-wise fashion, in which specific cortical regions multiple sclerosis patients present a correlation between cortical thinning (atrophy) over 2 years and anxiety severity (*p* < 0.05, controlling for multiple comparisons with Monte Carlo simulations). The resulting cluster, localized in the dorsal PFC, was then binarized and entered as seed to compute resting-state functional connectivity on a normative dataset [38]. Using the GSP normative dataset, we measured average blood–oxygen-level-dependent (BOLD) time courses within the seed corresponding to the anxiety-related atrophy map and correlated these values with the BOLD time course at every other brain voxel [30, 31]. This seed-based functional connectivity method is similar to lesion network mapping; the only difference is that instead of a brain lesion, the resulting atrophy map is used as a seed [44, 45]. Functional connectivity was determined by calculating the correlation between the mean time courses of the atrophied region of interest (ROI) and all other brain voxels in each of the 1000 images [45, 46]. The correlation values were then transformed to *z* values using the Fisher’s transform and used to compute a voxel-wise t-distribution that was finally thresholded at a voxel-wise family-wise error (FWE)-corrected value (*p* = 0.05). The connectivity maps were created in MNI space with 1.5 × 1.5 × 1.5 mm voxel size.

### Instructed threat paradigm

Before starting this investigation, participants were informed that one visual cue (circle) is associated with a mild electric shock with a probability of 33%, while the other visual cue (square) is safe. The intensity of the electric shock was calibrated for each subject, such that stimulation was highly fearful [minimum of seven on a scale of 0 (not fearful) to 10 (highly fearful)] [47]. The instructed threat paradigm (Fig. 4A) encompassed presenting on a computer screen the visual cue (circle or square) that denoted the anticipated condition (threat or safe). One second after every visual cue onset, a neuronavigated single-pulse TMS was applied on the right dorsomedial PFC. The stimulation intensity was 110% of resting-state motor threshold (RMT), as previously described [33]. Each trial consisted of presenting the visual cue on the screen for 5 s followed by a fixation cross on the center of the screen that jittered between 5 and 6 s. During the threat condition, electric shocks were applied to the dorsal part of the left hand with a probability of 33% with an electric stimulator (DS7A, Digitimer, USA) at any moment while the visual threat cue was present on the screen. In total, the paradigm consisted of 90 trials and lasted for 15 min. Continuous EEG recordings were performed in all participants for the complete duration of the paradigm. Furthermore, continuous resting-state EEG data was acquired in all participants for 5 min prior to the instructed threat paradigm, during which the participants were asked to sit still and think of nothing. The EEG data was acquired with a high-density (HD) 256 channel EEG system (Net Station 5.0, EGI, USA) operating at a sampling frequency of 250 Hz and electrode impedances below 50 kΩ [48].

### HD-EEG data processing

**Fig. 3:**
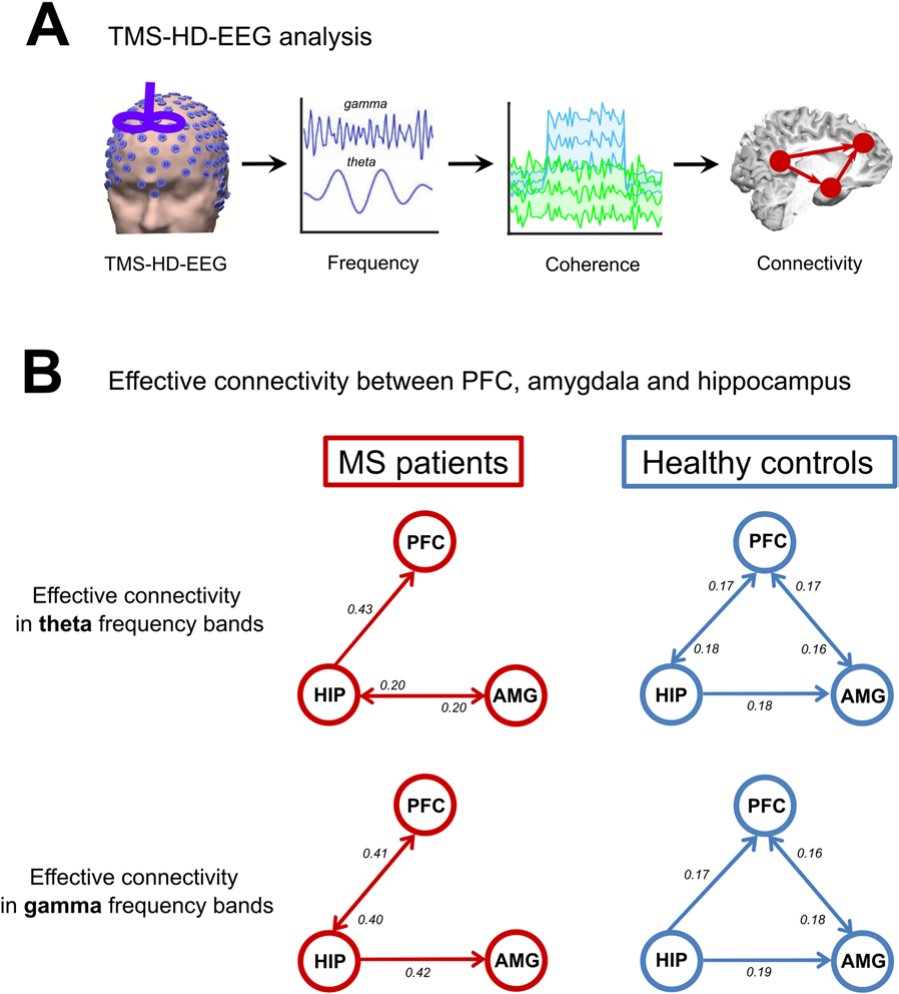
Impaired connectivity between anxiety-related regions in multiple sclerosis patients. **A** In a second patient cohort (*n* = 18) TMS–HD-EEG was performed at rest and effective connectivity between the PFC, amygdala and hippocampus was measured and compared to healthy controls (*n* = 18). **B** Theta and gamma frequency bands revealed no connectivity between PFC and amygdala in patients versus controls

The processing steps for HD-EEG data (Fig. 3A) were conducted in MATLAB R2015B (Mathworks, USA) using in-house customized analysis scripts and the open-source MATLAB toolbox Fieldtrip [49]. The continuous HD-EEG data acquired during the instructed threat paradigm was divided into epochs from − 2 to + 5 s relative to visual cue onset. Afterwards, the HD-EEG data from 0.005 s prior to and 0.02 s after the TMS pulse, which contained the TMS pulse itself and ringing artifacts, was removed. In addition, in all participants the trials in which electric shocks were administered were removed from further analysis. Then, HD-EEG data was re-referenced to a grand average of all electrodes. HD-EEG data was visually inspected and noisy trials were discarded. Subsequently, independent component analysis (ICA) was implemented and the components related to the physiological (eye blinks and muscle) and TMS (decay) artifacts were removed [50]. Finally, the remaining ICA components were transformed back into electrode data representation. The full description of the processing steps performed for resting-state HD-EEG data are described elsewhere [51]. Briefly, the continuous HD-EEG data was segmented into 2 s epochs after discarding the artifactual signals identified by visual inspection and ICA analysis. Afterwards, the source and connectivity analyses based on the power were performed in theta (4–7 Hz) and gamma (30–70 Hz) frequency bands.

### Heart rate estimation

The heart rate was extracted from the HD-EEG signals (Additional file 1: Fig. S1) using the extended version of the ICA algorithm, based on the information maximization [52] as previously reported [33].

### Source and connectivity analyses

The source analysis was conducted with the beamformer approach called dynamic imaging of coherent sources (DICS) for both resting state and instructed threat paradigm data in the theta and gamma frequency bands. The complete description of the analysis has been given elsewhere [53]. In brief, to determine the origin of HD-EEG activity in a specific frequency band observed over the scalp electrodes, both the forward problems and inverse problems need to be addressed. In this study, the lead-field matrix was modeled with the finite element method [54]. The DICS analysis was applied to extract the pooled source signals from three brain regions, namely right dorsomedial PFC, right amygdala and right hippocampus. These anatomical brain regions were defined according to our previous publication [33]. Finally, the connectivity fingerprints were extracted using the temporal partial directed coherence method (TPDC). The detailed description of the TPDC method has been previously described [54]. After estimating the TPDC values, the significance level was calculated from the applied data using a bootstrapping method [55]. In short, we divide the original time series into smaller non-overlapping windows and randomly shuffle the order of these windows to create a new time series. The MVAR (multivariate autoregressive) model is fitted to the shuffled time series and TPDC is estimated. The bootstrapping is performed 1000 times and the average TPDC value is taken as the significance threshold for all connections. The TPDC values were averaged across time [54]. This process is performed separately for each participant. In this study, the open-source MATLAB package autoregressive fit (ARFIT) [56, 57] was used for estimating the autoregressive coefficients from the spatially filtered source signals of the identified brain regions. We applied the time reversal technique [58] as a second significance test on the connections already identified by TPDC using a data-driven bootstrapping surrogate significance test.

### Structural equation modeling

SEM is an analytical tool used to determine causal relationships between variables in a model-based approach [59]. Here, the SEM analysis was performed using the SEM toolbox for MATLAB (https://www.mathworks.com/matlabcentral/fileexchange/60013-toolbox-for-structural-equation-modelling-sem) to assess the relationship between brain volumes (PFC, amygdala and hippocampus) and HADS-A score after 2 years.

We employed the maximum-likelihood method of estimation to fit the models. In order to adjust the models for a large sample size, we used the root mean square error of approximation (RMSEA) index, which improves precision without increasing bias [60]. The RMSEA index estimates lack of fit in a model compared to a perfect model and therefore should be low. Here, the RMSEA index for all models was below 0.05, implying a very good fit. In all models, the invariant under a constant scaling (ICS) and ICS factor criteria were close to zero, indicating that models were appropriate for analysis. Finally, using the Akaike information criterion (AIC) the quality of each model relative to other models was estimated, with smaller values signifying a better fit of the model. The obtained AIC comparing the models varied between 0.01 and 0.03 (good fit of the models). The strength of associations between the variables in the models was quantified by standardized coefficients (*s*), ranging from 0 (no association) to 1 (very strong association).

### Statistical analysis

The statistical analyses were performed with MATLAB R2015B and SPSS 23.0 (IBM, Armonk, NY, USA).

Vertex-wise regression analyses testing the association between cortical atrophy over 2 years with anxiety were assessed under the general linear model while adjusting for age and sex. Control for multiple comparisons was performed using Monte Carlo simulations (*n* = 1000, *p* < 0.05). *P* values < 0.05 were considered statistically significant.

To examine the significant differences in TPDC, two-tailed Student’s *t*-tests were performed. We performed a two-factorial ANOVA (groups, connections) for the TMS–HD-EEG connectivity analysis, separately for theta and gamma bands. Significant differences in oscillatory power were tested using nonparametric cluster-based statistics with the Monte-Carlo method in theta and gamma frequency bands [61].

## Results

### Cortical atrophy linked to anxiety symptoms

In order to identify anxiety-related regional changes in GM integrity, we investigated regional cortical and subcortical atrophy in a cohort of early multiple sclerosis patients over 2 years (Additional file 1: Tables S2 and S3) and anxiety symptoms assessed by the HADS-A scale (Fig. 1). Longitudinal cortical thickness analysis revealed widespread cortical thinning over the observation period in both hemispheres [left: *t* (91) = 4.4, *p* < 0.001; right: *t* (91) = 3.6, *p* = 0.001]. In the age- and sex-adjusted correlation analysis with the HADS-A score, we detected one prominent cluster: the left rostral middle frontal lobe, as part of the dorsal PFC was associated with HADS-A (*r* = 0.214; *p* = 0.040) (Fig. 2B, Additional file 1: Tables S4 and S5).

HADS-A scores in patients who developed a clinical relapse, EDSS-relevant progression or MRI activity (*n* = 57 patients, mean ± SD HADS-A score after 2 years = 5.2 ± 4.1) did not differ (independent Student’s *t* test: *p* = 0.441) from those patients who remained relapse free (*n* = 35 patients with NEDA-3, HADS-A score after 2 years = 5.9 ± 4.1).

### Anxiety-related atrophy network mapping

We hypothesized that anxiety-related cortical atrophy in multiple sclerosis patients would localize to a functional brain network. Hence, we applied a novel technique called atrophy network mapping to determine the brain regions functionally connected to the location of anxiety-related atrophy. Thus, binarized atrophy maps from the prior correlation analysis were used as seed points in functional connectivity analysis in a large (*n* = 1000) normative dataset. The resulting atrophy network map unveiled a specific network connectivity pattern (Fig. 2C) (statistical threshold *T* >  ± 15, corresponding to whole brain FWE-corrected *p* < 10^–12^) consisting of the ipsilateral PFC, but strikingly also the contralateral PFC and both amygdala and hippocampus. The functional network of other cortical or subcortical regions (e.g., basal ganglia) was not connected to the previously generated atrophy map.

### PFC, amygdala and hippocampus volumes predict anxiety symptoms

We next applied SEM to assess whether baseline volumes of the regions within the identified functional network, namely the PFC, amygdala and hippocampus, predict the severity of anxiety symptoms. The obtained fit indices in the SEM analysis implied a good fit of the constructed models to the observed data, providing robust relations between the variables. SEM revealed that the volumes of all three structures predict anxiety symptoms after 2 years in the 92 multiple sclerosis patients (Table 2). SEM with resultant standardized coefficients (*s*) identified the left amygdala (*s *= 0.852, *p* = 0.001) and the left hippocampus (*s* = 0.836, *p* = 0.002) as the strongest prognostic factors for anxiety severity 2 years after the initial MRI. The volumes of the PFC, amygdala and hippocampus derived from the cohort of multiple sclerosis patients who later underwent TMS–HD-EEG showed similar predictive powers for predicting anxiety symptoms (Table 2).

**Table 2:** Brain volumes and their capability in predicting anxiety through SEM

Anxiety-related brain structures	Anxiety (HADS-A) after 2 years (*n* = 92 patients)		Anxiety (HADS-A) within the TMS–HD-EEG cohort (*n* = 18 patients)	
	*s*	*p* value	*s*	*p* value
Amygdala (left)	0.852	0.001	0.886	0.001
Amygdala (right)	0.825	0.004	0.803	0.003
Hippocampus (left)	0.836	0.002	0.858	0.002
Hippocampus (right)	0.816	0.006	0.782	0.003
Prefrontal cortex (left)	0.675	0.007	0.604	0.010
Prefrontal cortex (right)	0.633	0.011	0.704	0.006

Association between brain volumes of the regions within the identified functional network and HADS-A score in the main cohort and the TMS–HD-EEG cohort. The predictive power is expressed as SEM-derived standardized coefficient (*s*)

*HADS-A* Hospital Anxiety and Depression Scale-Anxiety subscale, *TMS–HD-EEG* transcranial magnetic stimulation and high-density EEG, *SEM* structural equation modeling

### Reduced connectivity between PFC and amygdala in multiple sclerosis

**Fig. 4:**
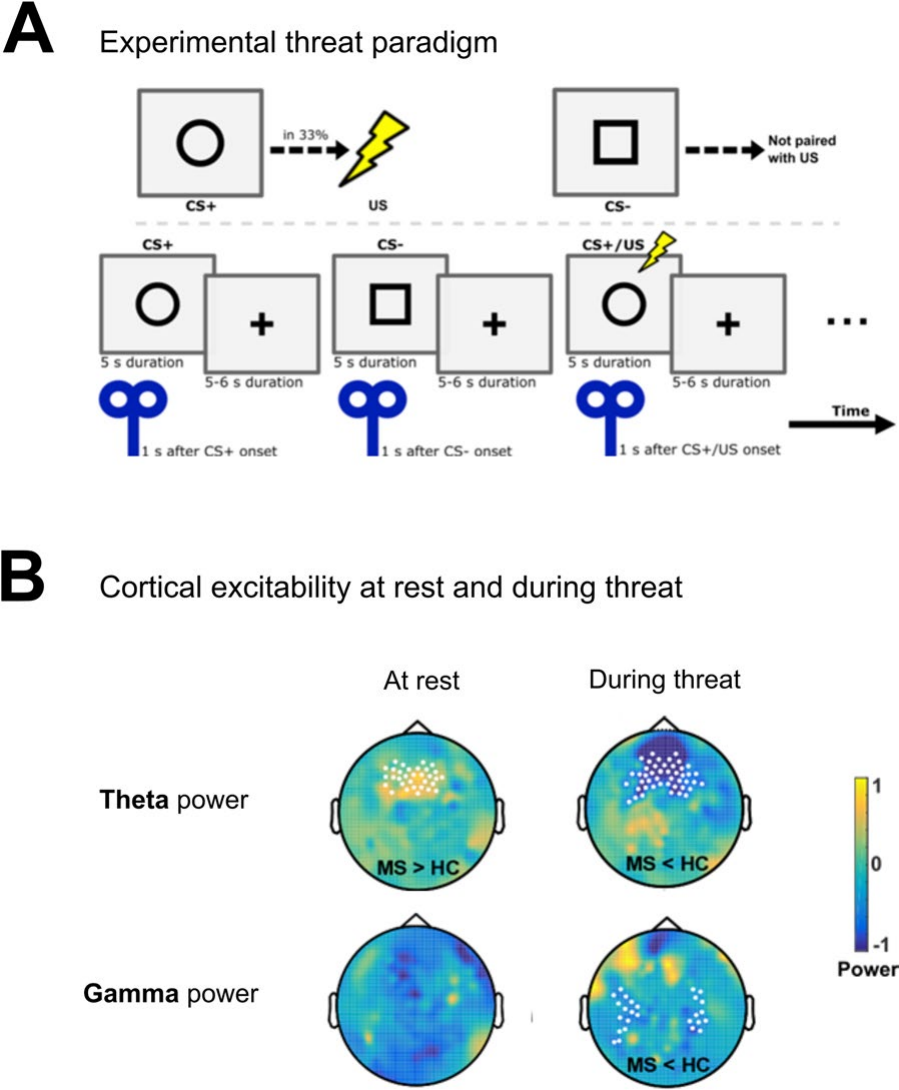
Altered cortical excitability in multiple sclerosis in response to threat. TMS–HD-EEG was performed in a second patient cohort (*n* = 18) and compared to healthy controls (*n* = 18). **A** Threat paradigm with a 33% chance of receiving a shock after appearance of a circle displayed on a monitor. A TMS pulse was applied 1 s after cue onset. HD-EEG (256 electrodes) was performed simultaneously. **B** Multiple sclerosis versus control showed an increased theta power at rest in prefrontal regions following TMS stimulation of the dorsal PFC; under threat, multiple sclerosis patients displayed a reduced theta and gamma power

To explore if the identified functional network from the atrophy network mapping approach is disturbed in multiple sclerosis patients, we investigated a cohort of 18 multiple sclerosis patients and 18 age-matched healthy controls in a TMS–HD-EEG experiment (Additional file 1: Table S1). Here, we first examined the effective connectivity between PFC, amygdala and hippocampus (Figs. 3 and 4) due to the results of the prior functional connectivity analysis findings in the main cohort. Strikingly, HD-EEG revealed differences in the theta and gamma frequency bands between the two groups for hippocampus-PFC, hippocampus-amygdala and PFC−amygdala connections at rest (Table 3, Additional file 1: Fig. S2A) and during task/threat paradigm (Table 3, Additional file 1: Fig. S2B, *p* < 0.01, two-sided *t* test).

**Table 3:** Coherence

Coherence	MS patients	Healthy controls	*p* value^a^
Theta (rest) HIP–PFC	0.33 ± 0.006	0.38 ± 0.05	0.0001
Gamma (rest) HIP–PFC	0.39 ± 0.04	0.22 ± 0.04	< 0.0001
Theta (after threat) HIP–PFC	0.41 ± 0.04	0.48 ± 0.05	0.0057
Gamma (after threat) HIP–PFC	0.32 ± 0.06	0.47 ± 0.04	0.0032
Theta (rest) HIP–AMG	0.39 ± 0.02	0.40 ± 0.05	0.132
Gamma (rest) HIP–AMG	0.45 ± 0.03	0.37 ± 0.02	< 0.0001
Theta (after threat) HIP–AMG	0.39 ± 0.05	0.50 ± 0.05	< 0.0001
Gamma (after threat) HIP–AMG	0.41 ± 0.03	0.29 ± 0.06	< 0.0001
Theta (at rest) PFC–AMG	0.40 ± 0.02	0.39 ± 0.03	0.234
Gamma (at rest) PFC–AMG	0.35 ± 0.03	0.44 ± 0.03	< 0.0001
Theta (after threat) PFC–AMG	0.42 ± 0.04	0.31 ± 0.01	< 0.0001
Gamma (after threat) PFC–AMG	0.22 ± 0.05	0.37 ± 0.04	< 0.0001

Coherence between prefrontal cortex, amygdala and hippocampus at rest and during threat processing in the TMS–HD-EEG study according to Additional file 1: Fig S2. The coherence is expressed as mean ± standard deviation

*PFC* prefrontal cortex, *HIP* hippocampus, *AMG* amygdala

^a^*P* values derived from two-tailed Student’s *t* test

Effective connectivity at rest between these three regions was different in multiple sclerosis patients as compared to healthy controls (Fig. 3). For the theta band, both the factor “group” [*F* (1, 244) = 268.82; *p* < 0.0001] and the factor “connection” [*F* (5, 244) = 14.42; *p* < 0.0001] significantly contributed to these alterations. The same was also true for the gamma band [factor “group”: *F* (1, 245) = 1029.9; *p* < 0.0001 and factor “connection”: *F* (4, 245) = 10.28; *p* < 0.0001]. All post hoc analyses were significant (*p* < 0.0001). Whereas the connectivity from hippocampus to PFC and amygdala was intact, we found a reduced connectivity between PFC and amygdala for the theta and gamma bands (*p* < 0.0001) (Fig. 3B) in multiple sclerosis patients. On the other hand, we found an increased information flow in the theta band in multiple sclerosis patients from hippocampus to PFC (*p* < 0.0001) possibly accounting for a compensatory mechanism. For the theta band, effective connectivity positively correlated with the PFC, amygdala and hippocampus volumes indicating in reverse that worse connectivity was associated with reduced volumes of these regions (Additional file 1: Table S6).

### Increased excitability in the PFC at rest

In order to investigate the potential pathophysiological cause underlying this disrupted information flow between the structures of the anxiety-related network, we assessed the HD-EEG power (hereinafter termed excitability) following a TMS pulse during an instructed threat paradigm. At rest, multiple sclerosis patients showed increased excitability compared to controls, evidenced as higher theta power in prefrontal electrodes after the TMS pulse at the dorsal PFC (Fig. 4B, *p* < 0.05, two-sided *t* test, 32 electrodes). This increase in the excitability was seen in the ipsilateral and contralateral PFC and detected up to 400 ms after the TMS stimulation.

Under threat condition, we found a decreased excitability (i.e. decreased theta power) (Fig. 4B, *p* < 0.05, two-sided *t* test, 45 electrodes) in the dorsal PFC, both after cue onset (0.15 s until 0.45 s) and after the TMS pulse (1.15 s until 1.45 s) in multiple sclerosis patients. These effects were seen during threat processing on both sides. Moreover, we observed a decreased gamma power for parieto-temporal electrodes in multiple sclerosis patients under threat condition (Fig. 4B, *p* < 0.05, two-sided *t* test, 26 electrodes). During the threat paradigm (Fig. 4A), the heart rate measured by beats per minute (bpm) of both multiple sclerosis patients (no threat: 73 ± 1.8 bpm; threat: 82 ± 1.5 bpm) and healthy controls (no threat: 72 ± 1.3 bpm; threat: 84 ± 1.6 bpm) increased (Additional file 1: Fig. S1, *p* < 0.05, two-sided *t* test), which served as a positive physiological control for our paradigm.

## Discussion

Our study provides evidence for functional network alterations underlying anxiety symptoms in multiple sclerosis. Using atrophy network mapping we identified a specific functional connectivity network consisting of the ipsi- and contralateral PFC as well as the amygdala and hippocampus. These brain areas are known to be essentially involved in emotion regulation in humans [62]. Structural equation modeling confirmed that the volumes of the PFC, amygdala and hippocampus were significant determinants that influence anxiety symptoms in multiple sclerosis. In a subsequent TMS–HD-EEG study, we found an impaired effective connectivity between the PFC and amygdala at rest in multiple sclerosis patients compared to healthy subjects. The underlying cause of this network disruption might be explained by an altered cortical excitability since we observed an increased excitability in prefrontal cortical regions in multiple sclerosis patients compared to controls at rest. Under threat, the PFC conversely showed a decreased excitability response in patients as compared to controls. Thus, altered neural excitability may underlie the observed disconnected network and may likewise underlie anxiety behavior as pathophysiological substrate [15].

### Structural correlates of multiple sclerosis-related anxiety

Structural and functional alterations of the PFC, amygdala and hippocampus have been reported in patients with anxiety disorders [63–70]. In contrast, structural or functional MRI correlates of anxiety symptoms in multiple sclerosis patients are still inconclusive, reflecting the complexity of the disease and challenges of available imaging technology [71, 72]. Whereas some early studies investigating multiple sclerosis-related anxiety showed no correlation with total lesion load or total brain volume [11, 12], recent advances in the imaging field now revealed an association of distinct regional lesion load measurements with anxiety [14, 73].

This ambiguity still leads to the notion that anxiety might be a reactive response following chronic disease progression (e.g., ongoing motor impairment) [11]. As a result of this, anxiety in multiple sclerosis is often classified as a mere comorbid condition [71]. However, our study provides evidence that anxiety symptoms in multiple sclerosis patients might be directly linked to its pathology, as patients scoring high on the HADS-A specifically exhibited increased atrophy in the PFC—a crucial area for top-down control for threat and emotional processing [32]. Although the official diagnosis of a generalized anxiety disorder according to the International Classification of Diseases would require a detailed personal interview, the HADS-A accurately identifies the presence of anxiety symptoms in multiple sclerosis. Moreover, the HADS-A has the highest sensitivity (82%) for detecting anxiety symptoms as compared to other anxiety scales in people with multiple sclerosis [74]. Notably, anxiety scores in our multiple sclerosis cohort were independent of relapse activity indicating that acute inflammatory activity was no confounder in the present study.

In a large meta-analysis of patients with various anxiety disorders, only atrophy of the anterior cingulate and inferior frontal cortex was associated with anxiety symptoms compared to healthy controls [69]. The anterior cingulate cortex is strongly connected with the PFC, a region that was identified in our study as being related to anxiety severity in multiple sclerosis. In addition, atrophy of the ventromedial PFC, a region associated with emotion and reward in decision-making, has been demonstrated in patients with generalized anxiety disorders [75]. Physiologically, activation of the medial PFC is associated with positive emotion, which can serve to regulate and diminish negative emotion [76]. In line with these results, our hypothesis is that the cortical thinning of the dorsal PFC in our study leads to impaired emotional processing, triggered by a network disruption that may increase the vulnerability for anxiety-related symptoms in multiple sclerosis.

### Networks related to anxiety in patients with multiple sclerosis

We observed a correlation between 2-year cortical thinning and anxiety severity solely in the left dorsal PFC. The peak region of anxiety-related cortical atrophy across multiple sclerosis patients was used as a seed location for functional connectivity analysis in normal healthy individuals [30]. This allowed us to identify a brain network functionally connected to atrophied locations [77]. The detected left-lateralized atrophy of the dorsal PFC was related to a network compromising the bilateral PFC, and—with the largest effects—the amygdala and hippocampus. Notably, these brain regions all play a prevailing role in processing threat and anxiety [63] and belong to the limbic system where subcortical structures meet the cerebral cortex [78].

The here identified anxiety-related network results in multiple sclerosis patients resemble previously observed patterns of network-level dysfunction described for generalized anxiety disorders [79]. The atrophy of the PFC, and hence the loss of structural cortical integrity, presumably alters the functional connectivity to specific brain areas (in this case, amygdala and hippocampus) distal from the primary spot of atrophy depicting the loss of information input from a damaged part of the brain [80].

Interestingly, in a recent meta-analysis including structural and functional MRI studies in generalized anxiety disorders, a reduced functional connectivity between PFC and amygdala was observed resulting from tasks investigating emotion dysregulation [67, 68]. Our fMRI and HD-EEG data acquired in multiple sclerosis patients supports this observation. The resulting impaired effective connectivity between the PFC and amygdala in our study is well in line with findings in anxiety development during adolescence [64] and is a replicated phenomenon in both the generation and regulation of emotions [65]. Furthermore, dysregulated prefrontal control over amygdala is engaged in the pathogenesis of anxiety disorders [66]. Here, we demonstrate in multiple sclerosis patients that through focal PFC atrophy the prefrontal control seems to become defective, resulting in aberrant amygdala activation and deficits in threat processing.

Additional evidence for the structure–function association between PFC, amygdala and hippocampus with anxiety development in multiple sclerosis was acquired by SEM. This predictive modeling is appropriate for employing complex models to evaluate hypothesized causal associations. The MRI volumes of all brain regions belonging to the detected functional network (PFC, amygdala and hippocampus) were associated with anxiety symptoms in multiple sclerosis. Interestingly, amygdala and hippocampus volumes of the left hemisphere showed a slightly higher predictive power than those of the right. This observation may prompt further investigations to address the issue of a possible lateralized involvement of these volumes implicated in limbic emotional circuits associated with anxiety.

### Network excitability at rest and during threat processing

Increased excitability of the PFC at rest as found here by TMS–HD-EEG, represents a compensatory mechanism for preservation of function (i.e. motor control) and is possibly due to locally reinforcing circuits [81]. Decreased excitability in the PFC upon threat in multiple sclerosis, however, indicates an impaired cortical processing under a stimulus, knowing that the PFC is normally activated during threat in healthy people [82]. In addition, we demonstrated that the effective connectivity from the PFC to the amygdala was specifically impaired in multiple sclerosis as compared to controls. These results provide evidence for a disturbed inhibitory role of the PFC on amygdala threat response in multiple sclerosis patients.

Therapeutically, repetitive TMS or transcranial direct current stimulation could be used to modulate brain networks through periodic treatments of preselected target areas. In fact, there are studies for multiple sclerosis using transcranial direct current stimulation to improve non-motor functions, such as fatigue [83–85] or cognition [86] with mild to moderate effects. These findings all support the involvement of impaired network synchronization in the disease pathology. In a recent study, impaired memory performance in multiple sclerosis patients was instantly restored via rebalancing impaired connectivity and excitability through targeted neuromodulation of the affected networks achieved by direct current or repetitive TMS of the dorsal PFC [18]. The latter strengthens our hypothesis that specific symptoms like anxiety can be referred to specific pathologic areas giving rise to functional network changes and thus be treated by normalizing the synchronization of brain oscillatory networks in multiple sclerosis.

## Conclusions

Our findings suggest that local anxiety-related structural changes in multiple sclerosis functionally spread beyond the sites of initial injury into widely interconnected areas and target a specific large-scale functional network that resembles known neurobiological anxiety circuits involving the PFC, amygdala and hippocampus. The here identified potential biological basis of anxiety in multiple sclerosis patients represents an opportunity for novel treatment approaches aiming to modulate brain networks in multiple sclerosis.

## Supplementary Information

**Additional file 1: Table S1.** Clinical data of the additional multiple sclerosis patients in the TMS–HD-EEG study. **Table S2.** Cortical volume changes over 2 years (ROI-wise). **Table S3.** Subcortical volume changes over 2 years. **Table S4.** Association between cortical atrophy over 2 years and HADS-A (anxiety) after 2 years. **Table S5.** Association between subcortical atrophy over 2 years and HADS-A (anxiety) after 2 years. **Table S6.** Correlations (*r*) between connectivity strength and volumes (prefrontal cortex, hippocampus and amygdala). **Figure S1.** Heart rate of multiple sclerosis patients and healthy controls during TMS–HD-EEG study. **Figure S2.** Connectivity/coherence. Connectivity and coherence between prefrontal cortex, amygdala and hippocampus at rest and during threat processing in the TMS–HD-EEG study.

## Data Availability

The data and algorithms essential to the conclusions of this study are available from the corresponding author upon reasonable request.

## Abbreviations

AIC: Akaike information criterion
BOLD: Blood–oxygen-level-dependent
DICS: Dynamic imaging of coherent sources
EDSS: Expanded Disability Status Scale
FLAIR: Fluid-attenuated inversion recovery
fMRI: Functional MRI
FEW: Family-wise error
FWHM: Full-width half-maximum
GM: Grey matter
GSP: Brain Genomics Superstruct Project
HADS-A: Hospital Anxiety and Depression Scale-Anxiety subscale
HD: High density
ICA: Independent component analysis
ICS: Invariant under a constant scaling
MNI: Montreal Neurological Institute
MP-RAGE: Magnetization-prepared rapid gradient echo
NEDA-3: No evidence of disease activity
PFC: Prefrontal cortex
RMSEA: Root mean square error of approximation
RMT: Resting-state motor threshold
ROI: Region of interest
SEM: Structural equation modeling
TMS–HD-EEG: Transcranial magnetic stimulation and high-density EEG
TPDC: Temporal partial directed coherence method
WM: White matter
