# Supplementary material for: Network alterations underlying anxiety symptoms in early multiple sclerosis
Source: J Neuroinflammation. 2022 May 24;19:119. doi: 10.1186/s12974-022-02476-0 (PMC9131528; doi:10.1186/s12974-022-02476-0)
Supplement: Supplementary file 1 — Additional file 1: Table S1. Clinical data of the additional multiple sclerosis patients in the TMS–HD-EEG study. Table S2. Cortical volume changes over 2 years (ROI-wise). Table S3. Subcortical volume changes over 2 years. Table S4. Association between cortical atrophy over 2 years and HADS-A (anxiety) after 2 years. Table S5. Association between subcortical atrophy over 2 years and HADS-A (anxiety) after 2 years. Table S6. Correlations (r) between connectivity strength and volumes (prefrontal cortex, hippocampus and amygdala). Figure S1. Heart rate of multiple sclerosis patients and healthy controls during TMS–HD-EEG study. Figure S2. Connectivity/coherence. Connectivity and coherence between prefrontal cortex, amygdala and hippocampus at rest and during threat processing in the TMS–HD-EEG study. [file 12974_2022_2476_MOESM1_ESM.docx]

**Additional file 1**

**Network alterations underlying anxiety symptoms in early multiple sclerosis**

Running title: Network alterations underlying anxiety

Erik Ellwardt, MD^1*^, Muthuraman Muthuraman, PhD^2*^, Gabriel Gonzalez-Escamilla, PhD^3^, Venkata Chaitanya Chirumamilla, PhD^3^, Felix Luessi, MD^1^, Stefan Bittner, MD^1^, Frauke Zipp, MD^1^, Sergiu Groppa, MD^3^, and Vinzenz Fleischer, MD^1^

| **Demographic and clinical data** | **Multiple sclerosis patients (n = 18)** |
| --- | --- |
| *Sex (female / male)* | 10/8 |
| *Disease course at baseline (CIS/RRMS)* | 3/15 |
| *Mean age at baseline MRI (SD) [years]* | 36.8 ± 9.4 |
| *Mean age at disease onset (SD) [years]* | 32.2 ± 8.9 |
| *Mean disease duration (SD) [years]* | 3.9 ± 5.0 |
| *DMD (no / first-line / second-line ^a)^* | 4/7/6 |
| *Mean EDSS score (SD)* | 2.2 ± 1.4 |
| *Mean HADS-A score (SD)* | 5.6 ± 2.9 |
| **Volumetric analysis** |  |
| *Mean GM volume (SD) [ml]* | 604 ± 73 |
| *Mean TB volume (SD) [ml]* | 1420 ± 113 |
| *Median T2 WM lesion volume (range) [ml]* | 2.0 (0.1 – 36.0) |

**Table S1.** **Clinical data of the additional multiple sclerosis patients in the TMS-HD-EEG study.** Demographic and clinical data as well as brain volumetric measurements of the additional cohort.

1. first-line: glatiramer acetate, interferon-beta, teriflunomide, dimethyl fumarate; second-line: natalizumab, fingolimod, alemtuzumab

**CIS** Clinically isolated syndrome

**RRMS**  Relapsing-remitting multiple sclerosis

**SD** Standard deviation

**EDSS**  Expanded Disability Status Scale

**GM** Grey matter

**TB** Total brain

**DMD** Disease-modifying drugs

**HADS-A** Hospital Anxiety and Depression Scale – Anxiety subscale

**Table S2.** Cortical volume changes over two years (ROI-wise).

| **Volume Brain Region** | Volume loss (ml) over two years  (n = 92 patients) | | |
| --- | --- | --- | --- |
|  | **Mean ± SD** | **T-value** | **p** |
| Bankssts (left) | 0.022 ± 0.082 | 2.5 | ***0.013*** |
| Caudal anterior cingulate (left) | 0.028 ± 0.154 | 1.7 | *0.085* |
| Caudal middle frontal (left) | 0.039 ± 0.111 | 3.3 | ***0.001*** |
| Cuneus (left) | 0.007 ± 0.077 | 0.8 | *0.421* |
| Entorhinal (left) | 0.035 ± 0.260 | 1.3 | *0.203* |
| Fusiform (left) | 0.033 ± 0.111 | 2.9 | ***0.005*** |
| Inferior parietal (left) | 0.026 ± 0.070 | 3.5 | ***0.001*** |
| Inferior temporal (left) | 0.025 ± 0.083 | 2.9 | ***0.004*** |
| Isthmus cingulate (left) | 0.048 ± 0.144 | 3.2 | ***0.002*** |
| Lateral occipital (left) | 0.019 ± 0.049 | 3.7 | ***<0.001*** |
| Lateral orbital frontal (left) | 0.013 ± 0.089 | 1.4 | *0.169* |
| Lingual (left) | 0.020 ± 0.077 | 2.4 | ***0.016*** |
| Medial orbital frontal (left) | -0.001 ± 0.090 | -0.1 | *0.077* |
| Middle temporal (left) | 0.023 ± 0.091 | 2.4 | ***0.017*** |
| Parahippocampal (left) | 0.018 ± 0.162 | 1.0 | *0.302* |
| Paracentral (left) | 0.017 ± 0.099 | 1.6 | *0.104* |
| Pars opercularis (left) | 0.027 ± 0.090 | 2.9 | ***0.005*** |
| Par orbitalis (left) | 0.015 ± 0.240 | 0.6 | *0.540* |
| Pars striangularis (left) | 0.022 ± 0.070 | 3.0 | ***0.003*** |
| Pericalcarine (left) | -0.001 ± 0.082 | -0.1 | *0.882* |
| Postcentral (left) | 0.025 ± 0.097 | 2.4 | ***0.017*** |
| Posterior cingulate (left) | 0.030 ± 0.120 | 2.4 | ***0.020*** |
| Precentral (left) | 0.037 ± 0.110 | 3.2 | ***0.002*** |
| Precuneus (left) | 0.031 ± 0.071 | 4.2 | ***<0.001*** |
| Rostral anterior cingulate (left) | 0.033 ± 0.127 | 2.5 | ***0.014*** |
| Rostral middle frontal (left) | 0.022 ± 0.065 | 3.3 | ***0.001*** |
| Superior frontal (left) | 0.039 ± 0.064 | 5.9 | ***<0.001*** |
| Superior parietal (left) | 0.026 ± 0.066 | 3.8 | ***<0.001*** |
| Superior temporal (left) | 0.037 ± 0.078 | 4.5 | ***<0.001*** |
| Supramarginal (left) | 0.024 ± 0.086 | 2.6 | ***0.010*** |
| Frontal pole (left) | 0.009 ± 0.218 | 0.4 | *0.704* |
| Temporal pole (left) | 0.041 ± 0.280 | 1.4 | *0.168* |
| Transverse temporal (left) | 0.008 ± 0.132 | 0.6 | *0.538* |
| Insula (left) | 0.038 ± 0.139 | 2.6 | ***0.011*** |
| Bankssts (right) | 0.030 ± 0.100 | 2.9 | ***0.004*** |
| Caudal anterior cingulate (right) | 0.009 ± 0.150 | 0.6 | *0.551* |
| Caudal middle frontal (right) | 0.017 ± 0.111 | 1.5 | *0.149* |
| Cuneus (right) | 0.012 ± 0.080 | 1.4 | *0.162* |
| Entorhinal (right) | 0.025 ± 0.272 | 0.9 | *0.377* |
| Fusiform (right) | 0.011 ± 0.111 | 0.9 | *0.363* |
| Inferior parietal (right) | 0.032 ± 0.0.86 | 3.6 | ***0.001*** |
| Inferior temporal (right) | 0.031 ± 0.117 | 2.5 | ***0.013*** |
| Isthmus cingulate (right) | 0.050 ± 0.217 | 2.2 | ***0.029*** |
| Lateral occipital (right) | 0.023 ± 0.083 | 2.6 | ***0.010*** |
| Lateral orbital frontal (right) | 0.031 ± 0.161 | 1.8 | *0.072* |
| Lingual (right) | 0.015 ± 0.106 | 1.4 | *0.172* |
| Medial orbital frontal (right) | 0.022 ± 0.099 | 2.1 | ***0.036*** |
| Middle temporal (right) | 0.029 ± 0.113 | 2.5 | ***0.015*** |
| Parahippocampal (right) | 0.029 ± 0.346 | 0.8 | *0.418* |
| Paracentral (right) | 0.031 ± 0.103 | 2.9 | ***0.005*** |
| Pars opercularis (right) | 0.024 ± 0.083 | 2.8 | ***0.006*** |
| Par orbitalis (right) | 0.024 ± 0.166 | 1.4 | *0.164* |
| Pars striangularis (right) | 0.012 ± 0.094 | 1.2 | *0.227* |
| Pericalcarine (right) | -0.006 ± 0.094 | -0.6 | *0.557* |
| Postcentral (right) | 0.023 ± 0.081 | 2.7 | ***0.009*** |
| Posterior cingulate (right) | 0.029 ± 0.106 | 2.7 | ***0.009*** |
| Precentral (right) | 0.024 ± 0.116 | 2.0 | *0.051* |
| Precuneus (right) | 0.034 ± 0.074 | 4.4 | ***<0.001*** |
| Rostral anterior cingulate (right) | 0.018 ± 0.157 | 1.1 | *0.278* |
| Rostral middle frontal (right) | 0.018 ± 0.074 | 2.3 | ***0.024*** |
| Superior frontal (right) | 0.033 ± 0.079 | 4.0 | ***<0.001*** |
| Superior parietal (right) | 0.041 ± 0.090 | 4.4 | ***<0.001*** |
| Superior temporal (right) | -0.002 ± 0.154 | -0.1 | *0.899* |
| Supramarginal (right) | 0.028 ± 0.086 | 3.2 | ***0.002*** |
| Frontal pole (right) | 0.057 ± 0.216 | 2.5 | ***0.013*** |
| Temporal pole (right) | 0.032 ± 0.333 | 0.9 | *0.365* |
| Transverse temporal (right) | 0.020 ± 0.232 | 0.8 | *0.408* |
| Insula (right) | 0.026 ± 0.137 | 1.8 | *0.073* |

**Table S3.** Subcortical volume changes over two years.

| **Volume Brain Region** | Volume loss (ml) over two years  (n = 92 patients) | | |
| --- | --- | --- | --- |
|  | **Mean ± SD** | **T-value** | **p** |
| Thalamus (left) | 143.3 ± 627.3 | 2.1 | ***0.037*** |
| Thalamus (right) | 125.6 ± 406.0 | 2.9 | ***0.005*** |
| Caudate (left) | 65.8 ± 165.4 | 3.7 | ***<0.001*** |
| Caudate (right) | 82.2 ± 145.8 | 5.2 | ***<0.001*** |
| Putamen (left) | 58.7 ± 324.7 | 1.7 | *0.097* |
| Putamen (right) | 75.7 ± 201.3 | 3.5 | ***0.001*** |
| Pallidum (left) | 58.4 ± 202.9 | 2.6 | ***0.009*** |
| Pallidum (right) | 21.3 ± 192.6 | 1.0 | *0.308* |
| Hippocampus (left) | 50.0 ± 198.2 | 2.3 | ***0.022*** |
| Hippocampus (right) | 59.8 ± 180.6 | 3.1 | ***0.003*** |
| Amygdala (left) | 32.4 ± 111.4 | 2.7 | ***0.009*** |
| Amygdala (right) | 0.6 ± 92.9 | 0.1 | *0.953* |
| Accumbens (left) | -14.1 ± 72.8 | -1.8 | *0.077* |
| Accumbens (right) | 11.9 ± 45.4 | 2.4 | ***0.017*** |
| CC Posterior | -2.2 ± 96.1 | -0.2 | *0.836* |
| CC Middle posterior | -3.4 ± 110.3 | -0.3 | *0.777* |
| CC Central | 25.4 ± 92.7 | 2.5 | ***0.013*** |
| CC Middle Anterior | 33.1 ± 91.7 | 3.3 | ***0.001*** |
| CC Anterior | 7.1 ± 92.4 | 0.7 | *0.475* |
| WM hypointensities | -271.7 ± 2047.2 | -1.2 | *0.222* |
| Cerebellum WM (left) | 217.8 ± 2689.1 | -0.7 | *0.455* |
| Cerebellum WM (right) | -129.4 ± 2380.1 | -0.5 | *0.616* |
| Cerebellum Cortex (left) | 879.5 ± 2735.8 | 3.0 | ***0.004*** |
| Cerebellum Cortex (right) | 787.3 ± 2275.3 | 3.2 | ***0.002*** |
| Brainstem | 233.2 ± 1019.8 | 2.1 | ***0.037*** |
| Brainstem Segment | 11760.8 ± 18091.4 | 6.0 | ***0.000*** |
| Cerebral WM (left) | 1145.2 ± 5319.8 | 2.0 | ***0.049*** |
| Cerebral WM (right) | 812.2 ± 6260.5 | 1.2 | *0.232* |
| Cerebral WM (total) | 1957.4 ± 11100.3 | 1.6 | *0.106* |
| Subcortical GM | 827.5 ± 1166.6 | 6.6 | ***<0.001*** |
| Total GM | 11334.0 ± 16511.7 | 6.4 | ***<0.001*** |
| Supratentorial | 10465.4 ± 16961.3 | 5.7 | ***<0.001*** |
| Total Brain | 3550.9 ± 16003.6 | 2.1 | ***0.043*** |

**Table S4.** Association between cortical atrophy over two years and HADS-A (anxiety) after two years.

| **Atrophy Brain Region** | Anxiety after two years  (n = 92 patients) | |
| --- | --- | --- |
|  | **r** | **p** |
| Bankssts (left) | 0.204 | *0.051* |
| Caudal anterior cingulate (left) | -0.152 | *0.147* |
| Caudal middle frontal (left) | 0.106 | *0.317* |
| Cuneus (left) | 0.125 | *0.234* |
| Entorhinal (left) | 0.060 | *0.571* |
| Fusiform (left) | 0.056 | *0.599* |
| Inferior parietal (left) | 0.106 | *0.313* |
| Inferior temporal (left) | 0.175 | *0.096* |
| Isthmus cingulate (left) | -0.078 | *0.461* |
| Lateral occipital (left) | 0.040 | *0.707* |
| Lateral orbital frontal (left) | 0.066 | *0.530* |
| Lingual (left) | 0.188 | *0.073* |
| Medial orbital frontal (left) | 0.033 | *0.754* |
| Middle temporal (left) | 0.116 | *0.269* |
| Parahippocampal (left) | 0.134 | *0.204* |
| Paracentral (left) | 0.000 | *0.999* |
| Pars opercularis (left) | 0.136 | *0.197* |
| Par orbitalis (left) | -0.007 | *0.946* |
| Pars striangularis (left) | 0.050 | *0.636* |
| Pericalcarine (left) | 0.172 | *0.100* |
| Postcentral (left) | 0.188 | *0.073* |
| Posterior cingulate (left) | 0.072 | *0.495* |
| Precentral (left) | 0.182 | *0.082* |
| Precuneus (left) | 0.117 | *0.266* |
| Rostral anterior cingulate (left) | -0.010 | *0.923* |
| Rostral middle frontal (left) | **0.214** | ***0.040*** |
| Superior frontal (left) | 0.119 | *0.260* |
| Superior parietal (left) | 0.201 | *0.054* |
| Superior temporal (left) | 0.185 | *0.077* |
| Supramarginal (left) | 0.106 | *0.316* |
| Frontal pole (left) | -0.157 | *0.136* |
| Temporal pole (left) | **-0.259** | ***0.013*** |
| Transverse temporal (left) | -0.065 | *0.540* |
| Insula (left) | -0.074 | *0.485* |
| Bankssts (right) | 0.095 | *0.366* |
| Caudal anterior cingulate (right) | 0.117 | *0.265* |
| Caudal middle frontal (right) | -0.084 | *0.428* |
| Cuneus (right) | 0.150 | *0.153* |
| Entorhinal (right) | -0.080 | *0.448* |
| Fusiform (right) | 0.015 | *0.889* |
| Inferior parietal (right) | 0.011 | *0.921* |
| Inferior temporal (right) | -0.058 | *0.580* |
| Isthmus cingulate (right) | 0.002 | *0.986* |
| Lateral occipital (right) | 0.066 | *0.531* |
| Lateral orbital frontal (right) | 0.081 | *0.442* |
| Lingual (right) | 0.166 | *0.113* |
| Medial orbital frontal (right) | 0.100 | *0.344* |
| Middle temporal (right) | -0.011 | *0.919* |
| Parahippocampal (right) | -0.087 | *0.411* |
| Paracentral (right) | -0.020 | *0.853* |
| Pars opercularis (right) | 0.114 | *0.277* |
| Par orbitalis (right) | -0.003 | *0.976* |
| Pars striangularis (right) | 0.058 | *0.582* |
| Pericalcarine (right) | 0.097 | *0.357* |
| Postcentral (right) | 0.120 | *0.255* |
| Posterior cingulate (right) | 0.112 | *0.287* |
| Precentral (right) | 0.075 | *0.478* |
| Precuneus (right) | 0.064 | *0.542* |
| Rostral anterior cingulate (right) | **-0.210** | ***0.045*** |
| Rostral middle frontal (right) | 0.064 | *0.542* |
| Superior frontal (right) | 0.051 | *0.630* |
| Superior parietal (right) | 0.051 | *0.630* |
| Superior temporal (right) | 0.117 | *0.268* |
| Supramarginal (right) | -0.006 | *0.952* |
| Frontal pole (right) | -0.086 | *0.414* |
| Temporal pole (right) | 0.104 | *0.323* |
| Transverse temporal (right) | 0.077 | *0.463* |
| Insula (right) | -0.111 | *0.292* |

| **Volume Brain Region** | Anxiety after two years  (n = 92 patients) | |
| --- | --- | --- |
|  | **r** | **p** |
| Thalamus (left) | -0.130 | *0.234* |
| Thalamus (right) | -0.150 | *0.169* |
| Caudate (left) | -0.048 | *0.660* |
| Caudate (right) | -0.151 | *0.165* |
| Putamen (left) | -0.062 | *0.569* |
| Putamen (right) | -0.056 | *0.611* |
| Pallidum (left) | -0.076 | *0.488* |
| Pallidum (right) | 0.002 | *0.987* |
| Hippocampus (left) | -0.116 | *0.287* |
| Hippocampus (right) | -0.200 | *0.065* |
| Amygdala (left) | -0.042 | *0.704* |
| Amygdala (right) | -0.121 | *0.269* |
| Accumbens (left) | -0.079 | *0.467* |
| Accumbens (right) | **-0.297** | ***0.006*** |
| CC Posterior | -0.068 | *0.533* |
| CC Middle posterior | 0.036 | *0.744* |
| CC Central | 0.017 | *0.874* |
| CC Middle Anterior | -0.033 | *0.760* |
| CC Anterior | 0.055 | *0.617* |
| WM hypointensities | -0.079 | *0.470* |
| Cerebellum WM (left) | 0.077 | *0.478* |
| Cerebellum WM (right) | -0.173 | *0.122* |
| Cerebellum Cortex (left) | -0.016 | *0.887* |
| Cerebellum Cortex (right) | 0.024 | *0.827* |
| Brainstem | 0.197 | *0.069* |
| Brainstem Segment | -0.065 | *0.554* |
| Cerebral WM (left) | -0.085 | *0.438* |
| Cerebral WM (right) | 0.023 | *0.836* |
| Cerebral WM (total) | -0.028 | *0.799* |
| Subcortical GM | **-0.242** | ***0.025*** |
| Total GM | -0.051 | *0.639* |
| Supratentorial | -0.056 | *0.611* |
| Total Brain | 0.012 | *0.910* |

**Table S5.** Association between subcortical atrophy over two years and HADS-A (anxiety) after two years.

**Table S6.** Correlations (r) between connectivity strength and volumes (prefrontal cortex, hippocampus and amygdala).

| Connectivity **to Brain Region Volume** | MS patients  (n = 18 patients) | | Healthy controls  (n = 18 patients) | |
| --- | --- | --- | --- | --- |
|  | **r** | **p** | **r** | **p** |
| Theta (rest) HIP - PFC **to PFC (left)** | 0.767 | *0.001* | -0.126 | *0.619* |
| Theta (rest) HIP - PFC **to PFC (right)** | 0.646 | *0.004* | 0.311 | *0.209* |
| Theta (task) HIP - PFC **to HIP (left)** | 0.591 | *0.01* | -0.102 | *0.687* |
| Gamma (rest) HIP - PFC **to PFC (right)** | -0.480 | *0.044* | -0.145 | *0.566* |
| Gamma (rest) HIP- AMG **to AMG (left)** | -0.019 | *0.941* | -0.496 | *0.036* |
| Gamma (rest) HIP- AMG **to AMG (right)** | 0.111 | *0.660* | -0.570 | *0.014* |
| Gamma (rest) HIP - AMG **to PFC (right)** | -0.180 | *0.474* | 0.557 | *0.016* |
| Gamma (task) HIP - AMG **to AMG (left)** | -0.517 | *0.028* | 0.576 | *0.012* |
| Gamma (task) PFC - AMG **to HIP (right)** | n. a. | *n. a.* | 0.492 | *0.038* |

**PFC** Prefrontal cortex

**HIP**  Hippocampus

**AMG** Amygdala

**Figure S1:** **Heart rate of multiple sclerosis patients and healthy controls during TMS-HD-EEG study.** Increase in heart rate after threat onset compared to non-threat conditions. (**p < 0.01, two sided student t-test).

**
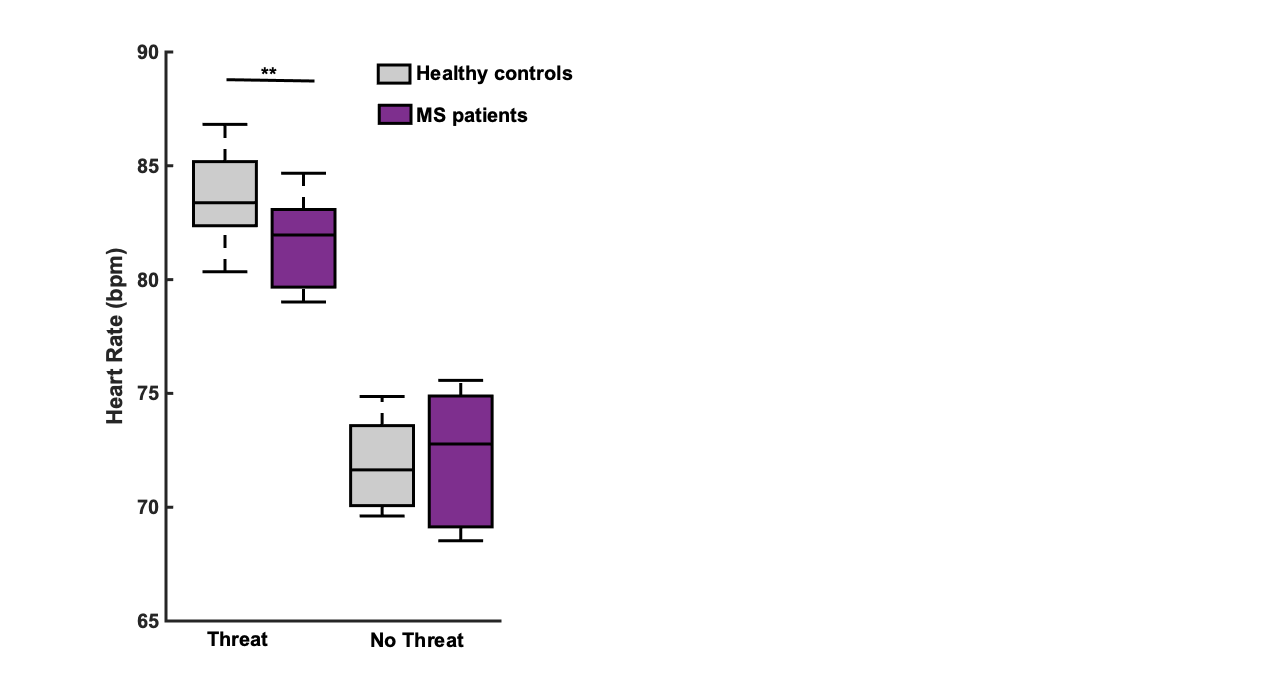
**

**Figure S2. Connectivity / coherence.** Connectivity and coherence between prefrontal cortex, amygdala and hippocampus at rest and during threat processing in the TMS-HD-EEG study.

**
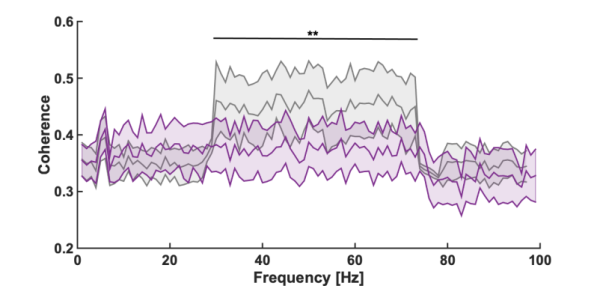

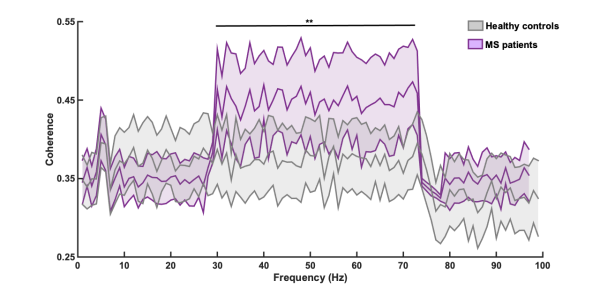

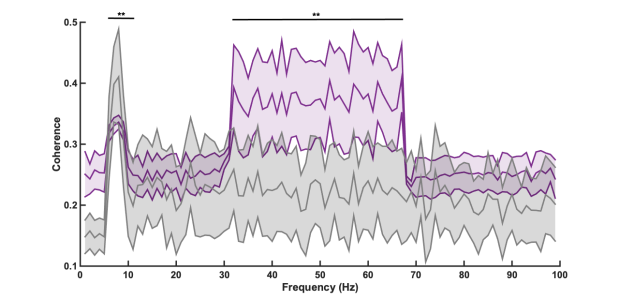
**

**B After threat**

**AMG**

**HIP**

**A At rest**

**
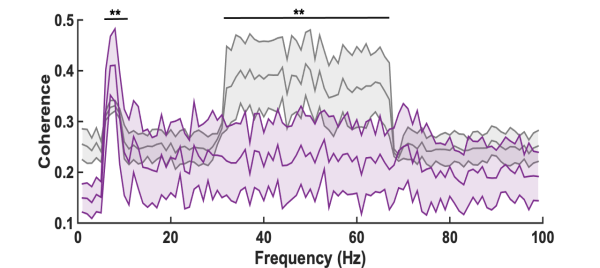

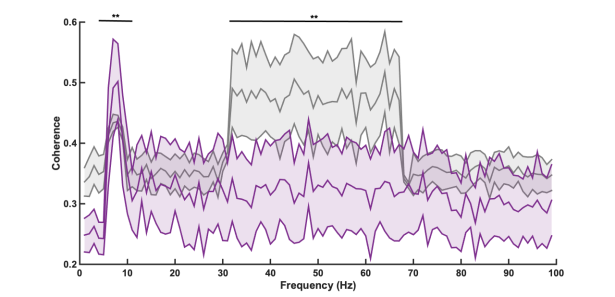

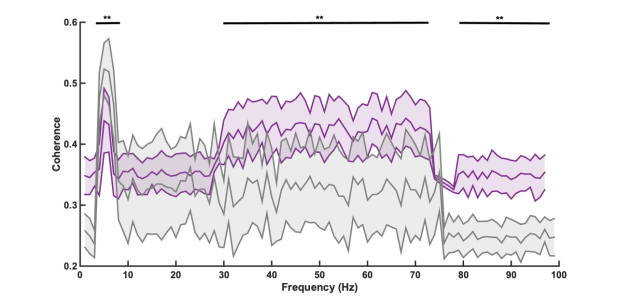
**

**HIP**

**AMG**

**PFC**

**PFC**
